# Dual regime of flowering time and pollination enhances pollen tube development in *Ziziphus*

**DOI:** 10.1093/hr/uhag066

**Published:** 2026-03-07

**Authors:** Qingjun Li, Xiaoning Zheng, Jiurui Wang, Qiong Zhang, Ning Wang, Yinming Li, Mengjun Liu

**Affiliations:** College of Horticulture/Research Center of Chinese Jujube, Hebei Agricultural University, Baoding, Hebei 071001, China; Pomology Institute, Binzhou Academy of Agricultural Sciences, Binzhou, Shandong 256600, China; Wudi Inspection and Testing Center, Binzhou, Shandong Province 251900, China; College of Horticulture/Research Center of Chinese Jujube, Hebei Agricultural University, Baoding, Hebei 071001, China; Shandong Institute of Pomology, Taian, Shandong 271000, China; Pomology Institute, Binzhou Academy of Agricultural Sciences, Binzhou, Shandong 256600, China; College of Foreign Language, Hebei Agricultural University, Baoding, Hebei 071001, China; College of Horticulture/Research Center of Chinese Jujube, Hebei Agricultural University, Baoding, Hebei 071001, China

## Abstract

Chinese jujube (*Ziziphus jujuba* Mill., 2*n* = 2*x* = 24) is a drought-tolerant, nutrient-rich fruit crop. However, its genetic improvement is constrained by protandry, low fruit set, and severe embryo abortion. Interspecies hybridization between Chinese jujube and Indian jujube (*Ziziphus mauritiana* Lam., 2*n* = 4*x* = 48) is further hindered by asynchronous flowering. We developed a dual-regime protocol combining temperature control and strategic heavy pruning to advance the flowering time of Indian jujube (cultivar ‘Niunaidaqingzao’, N) by 2 months, thereby synchronizing its anthesis with that of Chinese jujube (‘Dongzao’, D) and wild Chinese jujube (‘Suanzao’, S). *In vitro* artificial self-pollination (AS) and *in vitro* artificial cross-pollination (AC) were conducted to assess pollen tube elongation and ovary expansion. Triple AS (TAS) boosted pollen tube emergence to 59%–87% across the three genotypes, more than doubling *in vitro* spontaneous self-pollination (SSP*)* rates and outperforming single AS 1.4 to 2.7 times (*P* < 0.05). Ovary-swelling frequencies of TAS reached 68.52% in wild Chinese jujube S and 27.78% in Indian jujube N, indicating 2.85 and 2.14 times increases over SSP and 1.88–4.11 times increases over single AS. In ♀S × ♂D, ♀D × ♂S, and ♀S × ♂N crosses, triple AC (TAC) raised pollen tube emergence to 54%–72% (1.3–2.2 times of single AC) and ovary expansion to 26%–39% (1.4–1.9 times of single AC) (*P* < 0.05). These findings provide a practical and efficient strategy for overcoming asynchronous flowering and reproductive barriers of interspecies hybridization in genus *Ziziphus*, enabling the establishment of interspecies hybrid populations for downstream breeding programs.

## Introduction

Chinese jujube (*Ziziphus jujuba* Mill.), an ancient cultivated fruit tree indigenous to the middle–lower reaches of the Yellow River in China, has been cultivated and utilized for over 7000 years. Its wild ancestor, wild Chinese jujube Suanzao, contains pharmacologically active compounds such as jujubosides and spinosin, which exhibit sedative and tonic effects [1, 2]. Introduced to more than 40 countries, Chinese jujube provides economic and ecological value [3]. Compared with other fruit crops, jujube breeding remains significantly underdeveloped [4], representing a critical bottleneck for industry-wide quality improvement.

At present, there are two urgent and high-impact objectives that define jujube breeding. First, cultivars must be tailored to specific end uses [5]. Based on fruit traits and phytochemical profiles, jujube cultivars fall into four categories: fresh-eating, dried, medicinal, and processing types [6]. Developing high-functional and distinctive cultivars is essential for advancing the jujube industry [7]. Second, superior new cultivars must exhibit strong resistance to pests and diseases. Jujube witches’ broom disease and fruit cracking have long constrained production [8–10]. Therefore, breeding cultivars that combine high resistance with high yield, superior quality, and ease of management represents a historic imperative for the industry [11].

Jujube breeding faces several practical challenges. The flowers are small, with corollas measuring barely 5 mm in diameter, making manual emasculation exceptionally difficult. Even slight mechanical damage often leads to floral abscission. Natural fruit set in jujube is extremely low—approximately 1% and embryonic abortion rates are very high [12]. Consequently, the recovery rate of hybrids from controlled crosses typically falls below 0.01% [13]. Autopolyploid induction and net-cage combined with honeybee-assisted pollination have yielded incremental improvements [12, 14]. However, autopolyploid induction cannot introduce exotic alleles for multitrait pyramiding. Meanwhile, net-cage and honeybee-assisted pollination is limited by regional and phenological mismatches, low efficiency, and high costs. Compounding these challenges, an efficient genetic engineering system for jujube has yet to be established [15].

Interspecies hybridization between Chinese jujube and Indian jujube opens a new avenue for jujube breeding. Chinese jujube is renowned for its fruit’s high sugar content, vitamin C, cyclic nucleotides, and exceptional drought and cold tolerance. Indian jujube combines resistance to jujube witches’ broom disease and fruit cracking with large fruit size, high yield, and medicinal properties such as sedative, hemopoietic, and digestive benefits. Therefore, progenies from interspecific crosses are expected to exhibit enlarged fruit, enhanced pest and disease resistance, and increased genetic diversity in Chinese jujube [16]. However, a major barrier is asynchronous flowering. Under field conditions, Chinese jujube grown in northern China blooms from May to July, whereas Indian jujube cultivated in southern China does not bloom until at least August. Even when Indian jujube is transplanted into greenhouses in northern China, a marked temporal gap still exists between their blooming periods. Attempts to store Chinese jujube pollen at 4°C until July failed, as pollen viability was completely lost after 2 months of cold storage [17].

In this study, we integrated temperature control with different pruning methods to shift the flowering time of Indian jujube N to April–July, achieving complete synchrony with two Chinese jujube cultivars. *In vitro* culture of parental flowers combined with triple artificial pollination overcame the incompatibility barriers in both intraspecific and interspecific crosses within the genus *Ziziphus*, promoting consistent pollen tube germination and elongation on the stigma as well as enlargement of the fertilized ovary. This strategy establishes an efficient pathway for interspecies hybridization in the genus *Ziziphus* and provides a technical foundation for breeding superior cultivars.

## Results

### Synchronization of the flowering time of Chinese jujube and Indian jujube

**Figure 1:**
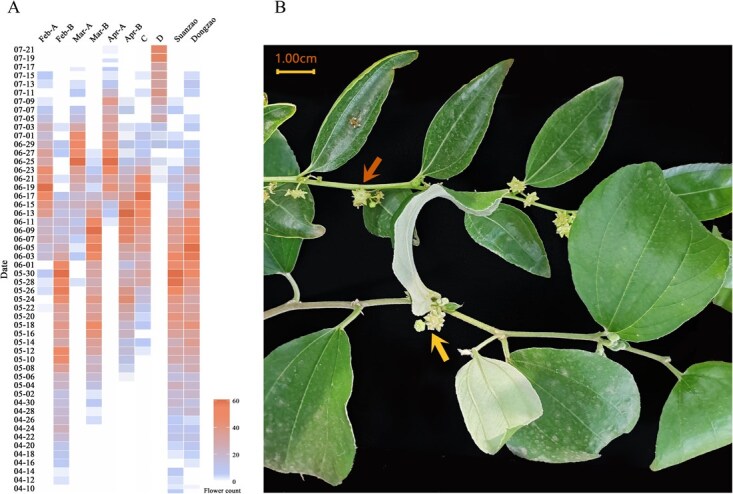
Advancement of the flowering time of Indian jujube N to April to achieve synchronization with Chinese jujube. (A) Feb-A, Mar-A, and Apr-A denote heavy pruning and retaining five to six primary branches in February, March, and April, respectively, under controlled ambient temperature, with no secondary branches retained. Feb-B, Mar-B, and Apr-B denote heavy pruning of primary branches in February, March, and April, respectively, under the same temperature conditions, retaining five to six primary branches each with only one secondary branch. C (CK1) involved temperature control only; D (CK2) involved neither temperature control nor pruning. (B) Under the Feb-B treatment, Indian jujube N and the Chinese jujube D flowered simultaneously in April. The arrow in the upper part indicates the opened flower of Chinese jujube D; The arrow in the lower part indicates the opened flower of Indian jujube N.

In greenhouse 1 (with low-temperature treatment), treatment B (Feb-B, retaining five to six primary branches each with only one secondary branch) advanced the first bloom of Indian jujube N to 12 April, ~2 months earlier than that of CK2 (no pruning, no temperature control) in greenhouse 2. This timing coincided with the flowering time of Wild Chinese jujube S and Chinese jujube D. Repeating treatment B on 14 March (Mar-B) and 1 April (Apr-B) advanced the first bloom to 27 April and 7 May, respectively. In contrast, treatment A (retaining only five to six primary branches without preserving any secondary branches) delayed flowering by 30–40 days relative to treatment B. Between late February and early April, the earlier the heavy pruning was performed, the greater the advancement in flowering time. In the winter-heated solar greenhouse 2, CK2 (treatment D) remained in vegetative growth until mid-June. These results indicate that the synergistic effect of temperature control and proper pruning can effectively regulate the flowering time of Indian jujube N (Fig. 1).

### Comparison of the single flower opening process between Chinese jujube and Indian jujube

**Figure 2:**
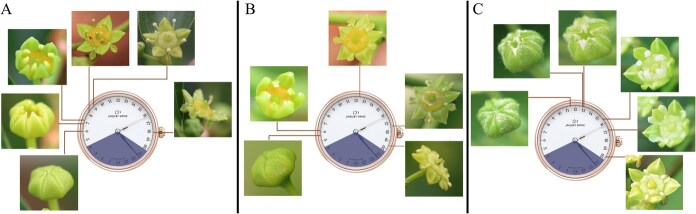
The flowering process and timing of the wild Chinese jujube, Chinese jujube, and Indian jujube.

The single flower opening process in *Ziziphus* was divided into five sequential stages: (i) bud dehiscence, (ii) calyx and petal expansion, (iii) stamen extension and expansion, (iv) stigma protrusion and reflexing, and (v) flower senescence. During stage (i), filaments transitioned from incurved to erect; in stage (ii), petals flattened; in stage (iii), filaments fully extended; stage (iv) coincided with stigma emergence and splitting; and stage (v) was characterized by wilting of the stigma and anthers. Flowering phenology varied between genotype and species. Chinese jujube S: Flower dehiscence occurred between 05:30 and 06:30, which was followed by calyx and petal expansion from 06:30 to 08:00. Stamen extension and expansion took place from 08:00 to 10:00. Stigma protrusion began approximately 1.5 hours later than that of dehiscence (around 07:00–07:30). Stigma tip separation occurred after 09:00, with reflexing observed after 13:00.For Indian jujube N, flower bud dehiscence occurred from 11:30 to 12:40, calyx and petal expansion was from 12:40 to 17:30, stamen extension and stigma protrusion was from 17:30 to 21:00, and senescence after 21:00.For Indian jujube N, flower bud dehiscence occurred from 11:30 to 12:40, calyx and petal expansion from 12:40 to 17:30, stamen extension and stigma protrusion from 17:30 to 21:00, and senescence after 21:00 (Fig. 2). All their pollen viability peaked during calyx and petal expansion [stage (ii)]. Meanwhile, stigma receptivity—defined as the capacity to support pollen germination—peaked when the stigma was protruding and dehiscing and was covered with a clear, viscous exudate [18], spanning stages (iii) and (iv). This finding is highly significant for optimizing pollen collection and pollination timing to enhance hybridization success. These observations demonstrate that anthesis is asynchronous among species and genotypes; within individual flowers, pollen maturation and stigma receptivity do not overlap well. Therefore, whether for self-pollination or hybridization, precise and effective pollination requires specific interventions—such as collecting pollen at peak viability from the male parent, storing it at 4°C, and pollinating the female parent once the stigma has reached maturity.

### Comparison of the pollen viability between Chinese jujube and Indian jujube

**Figure 3:**
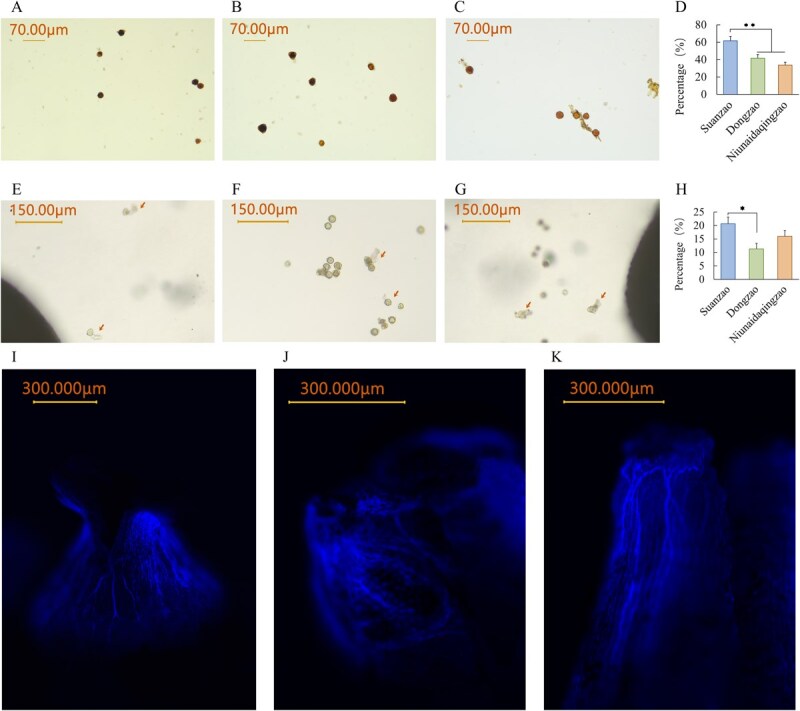
Pollen viability and self-pollinated pollen tube elongation. (A) Pollen viability of wild Chinese jujube S. (B) Pollen viability of Chinese jujube D. (C) Pollen viability of Indian jujube N. (D) Statistical chart of pollen viability for wild Chinese jujube S, Chinese jujube D, and Indian jujube N. (E) Wild Chinese jujube S pollen germination. (F) Chinese jujube D pollen germination. (G) Indian jujube N pollen germination. (H) Pollen germination statistics chart for wild Chinese jujube S, Chinese jujube D, and Indian jujube N. (I–K) Self-pollinated pollen tube elongation in wild Chinese jujube S, Chinese jujube D and Indian jujube N.

Two approaches were used to assess pollen viability. The I₂-KI staining method revealed peak viability at 2–2.5 h after flower bud dehiscence in wild Chinese jujube S, 2–4 h in Chinese jujube D, and 3.5–5 h in Indian jujube N (Table S1), with values of 61% ± 5.06%, 42% ± 4.16%, and 34% ± 3.40% (Table S2), respectively. *In vitro* germination assays conducted under the same conditions yielded germination rates of 20.68% (Chinese jujube S), 11.33% (Chinese jujube D), and 16% (Indian jujube N) (Fig. 3, Table S3). These results provide a basis for evaluating crossing potential.

### Interspecies and intraspecies *in vitro* pollination and ovary expansion

**Figure 4:**
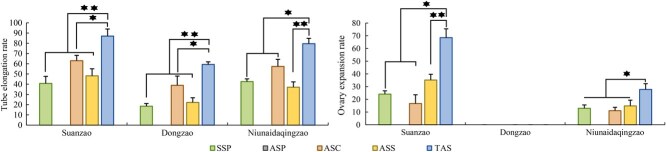
Pollen tube elongation and ovary expansion rate of *in vitro* self-pollination. SSP: *in vitro* SSP. ASP: single *in vitro* artificial self-pollination performed before stigma separation. ASC: single *in vitro* artificial self-pollination at the stigma separation stage. ASS: *in vitro* artificial self-pollination after the stigma has reflexed outward. TAS: triple *in vitro* artificial self-pollination.

Although protandry causes asynchrony between pollen and stigma maturation, some pollen from wild Chinese jujube S, Chinese jujube D, and Indian jujube N germinated on self-stigmas (Fig. 3, Table S4); however, no ovary expansion was observed. This may be attributed to pollen aging and the associated decline in viability. Triple *in vitro* artificial self-pollination (TAS, Table 1) effectively promoted pollen germination and ovary expansion. TAS achieved pollen tube elongation rates of 87.4% in Chinese jujube S, 59.26% in Chinese jujube D, and 79.63% in Indian jujube N, representing 2.13, 3.2, and 1.8 times increases compared to *in vitro* spontaneous self-pollination (SSP, Table 1), respectively (*P* < 0.05). Compared with single AS, TAS resulted in 1.38–2.67 times higher pollen tube elongation rates *(P* < 0.05), whereas single *in vitro* artificial self-pollination performed before stigma separation (ASP, Table 1) resulted in no pollen tube elongation in each of the tested Chinese jujube or Indian jujube genotypes. None of the AS treatments induced ovary expansion in Chinese jujube D, confirming its gametophytic self-incompatibility [19, 20]. In wild Chinese jujube S and Indian jujube N, AS produced ovary expansion rates of 68.52% and 27.78%, respectively, significantly higher than those under SSP (24.07% and 12.96%) and single AS (16.67%–35.18% and 11.11%–14.81%) (Fig. 4, Table S5).

**Table 1:** Treatment design.

**Treatments**	**Processing settings**
SSP	*In vitro* SSP.
AS	*In vitro* artificial self-pollination
ASP	*In vitro* artificial self-pollination was conducted when the stigma had emerged but had not yet separated
ASC	*In vitro* artificial self-pollination was conducted at the stage when the stigma had separated
ASS	*In vitro* artificial self-pollination was performed at the stage when the stigma had separated and curled outward
TAS	*In vitro* triple artificial self-pollination was performed at three developmental stages: prior to stigma protrusion, during protrusion but before separation, and after separation with outward curling
AC	*in vitro* artificial cross-pollination
ACP	*In vitro* artificial cross-pollination was conducted at the stage when the stigma was protruding but had not yet separated
ACC	*In vitro* artificial cross-pollination was conducted at the stage when the stigma had separated
ACS	*In vitro* artificial cross-pollination was performed at the stage when the stigma had separated and curled outward
TAC	*In vitro* triple artificial cross-pollination was performed at three distinct stages: prior to stigma protrusion, during stigma protrusion but before separation, and after stigma separation and outward curling

**Figure 5:**
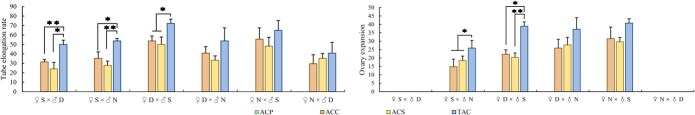
Pollen tube elongation and ovary expansion rate. ACP: single *in vitro* artificial cross-pollination performed before stigma separation. ACC: single *in vitro* artificial cross-pollination at the stigma separation stage. ACS: *in vitro* artificial cross-pollination after the stigma has reflexed outward. TAC: triple *in vitro* artificial cross-pollination.

Similarly, triple *in vitro* artificial cross-pollination (TAC, Table 1) enhanced crossing efficiency. In the three crossing combinations ♀S × ♂D, ♀S × ♂N, and ♀D × ♂S, TAC achieved significantly higher pollen tube elongation rates of 53.70%, 72.22%, and 53.70%, representing 1.34–2.23 times increases compared to single AC (*P* < 0.01) (Table S6). Among the six possible reciprocal crosses, ovary expansion occurred in four crosses (♀S × ♂N, ♀D × ♂S, ♀D × ♂N, and ♀N × ♂S). In ♀S × ♂N, TAC produced an ovary expansion rate of 25.93%, 1.40–1.75 times higher than single AC, while in ♀D × ♂S, it reached 38.89%, 1.91–1.92 times higher than single AC (*P* < 0.05). No ovary expansion occurred when Chinese jujube D was used as the male parent (♀S × ♂D and ♀N × ♂D) (Fig. 5).

**Figure 6:**
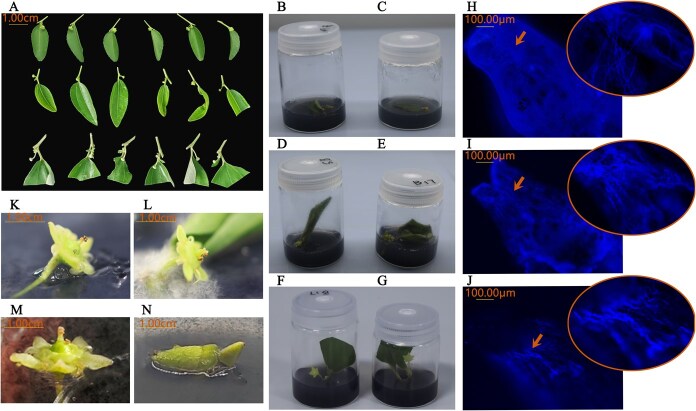
Flowchart of *in vitro* hybridization. (A) Parental flower buds with leaf used for *in vitro* hybridization. (B) *In vitro* culture of ♀S × ♂N cross. (C) *In vitro* culture of ♀S × ♂D cross. (D) *In vitro* culture of ♀D × ♂N cross. (E) *In vitro* culture of ♀D × ♂S cross. (F) *In vitro* culture of ♀N × ♂S cross. (G) *In vitro* culture of ♀N × ♂D cross. (H) Pollen tube elongation in ♀N × ♂S at 24 h postpollination. (I) Pollen tube elongation in ♀D × ♂S at 24 h postpollination. (J) Pollen tube elongation in ♀D × ♂N at 24 h postpollination. (K) Ovary expansion in ♀N × ♂S at 5 days postpollination. (L) Ovary expansion in ♀D × ♂S at 5 days postpollination. (M) Ovary expansion in ♀D × ♂N at 5 days postpollination. (N) Ovary expansion in ♀D × ♂N at 16 days postpollination.

Across the six interspecific and intraspecific combinations, no pollen tubes were observed to penetrate the stigma in ASP and ACP. (*In vitro* artificial cross-pollination was conducted at the stage when the stigma was protruding but had not yet separated, Table 1.) In the three combinations, ♀S × ♂D, ♀S × ♂N, and ♀D × ♂S, the pollen tube elongation rate in the TAC was significantly higher (1.34–2.23 times) than that in the single AC after stigma separation (Fig. 5). Ovaries exhibited swelling following pollination in the four combinations, ♀S × ♂N, ♀D × ♂S, ♀D × ♂N, and ♀N × ♂S, with TAC yielding the highest swelling rates. In the ♀S × ♂N combination, the ovary expansion rate under TAC was 40% and 75% higher than in the single AC, respectively. In the ♀D × ♂S combination, the ovary expansion rate under TAC was 91% and 92% higher than in the single AC, respectively. All these differences were statistically significant. In contrast, no ovary enlargement occurred in the two combinations using Chinese jujube D as the male parent (♀S × ♂D and ♀N × ♂D) (Fig. 5). Therefore, Chinese jujube D is unsuitable as a male parent for hybridization. These results further confirm the critical role of TAC in enhancing hybridization success rates (Fig. 6, Table S7).

## Discussion

Self-incompatibility and cross-incompatibility operate across morphological, physiological, genetic, molecular, and environmental scales [21, 22], preventing inbreeding depression while maintaining species boundaries. In jujube, these barriers include gametophytic self-incompatibility [20], protandry, and structural floral isolation. Anatomical and field observations revealed that in wild Chinese jujube S, Chinese jujube D, and Indian jujube N, stamens dehisce before pistil maturation. This temporal mismatch prevents pollen release from coinciding with stigma receptivity [23–25]. In this study, the fact that only a small amount of pollen germinated on the stigma after SSP, without any ovary expansion, further corroborates this temporal mismatch. Under TAS, the proportion of ovary expansion in wild Chinese jujube S and Indian jujube N was significantly higher than that in the other treatments. Pollen tube elongation following single *in vitro* artificial self-pollination at the stigma separation stage (ASC, Table 1) was significantly higher than that following SSP or single *in vitro* artificial self-pollination after the stigma has reflexed outward (ASS, Table 1). This indicates that the processes of pollen attachment, hydration, and germination on the stigma are strictly dependent on stigma status [26]. Successful fertilization requires a sufficiently mature stigma capable of supporting pollen germination. A freshly separated stigma is essential for effective pollen tube germination and growth. Before separation, the stigma is functionally incompetent; after reflexing, it rapidly senesces and loses receptivity. Consequently, timely pollen supplementation at the stigma separation stage could maximize pollen tube elongation [27]. Exploiting protandry through precise understanding of stamen–pistil ontogeny eliminates the need for emasculation in scheduled jujube breeding programs, providing a theoretical foundation for optimizing pollination timing and enhancing hybridization success.

Chinese jujube exhibits small flowers and low pollen production [28]. Both stigmas and flowers are short lived [29, 30]. Reproductive success, therefore, depends on the accurate and timely deposition of a sufficient quantity of high-quality pollen onto the stigma surface [31, 32]. In this study, triple artificial pollination increased the pollen tube elongation rate to 1.8–3.2 times that of spontaneous pollination, comparable to the two-step pollination efficiency reported in *Arabidopsis* [33]. Enhanced reproductive success in jujube may be attributed to increased pollen deposition and reactivation of stigma receptors. Each doubling of pollen deposition could increase ovule-targeting success by ~40% [34]. Triple pollination provides additional opportunities for a greater number of superior pollen tubes to reach the embryo sac. Sequential pollen loads also reprimed partially desensitized stigma receptors, triggering a new round of callose degradation, thereby widening the pollen tube entry window and significantly enhancing fertilization recovery [35, 36]. The temporal dispersion inherent in triple artificial pollination staggered and overlapped the viability peaks of successive pollen batches, ensuring fertilization under optimal microenvironmental conditions. Consequently, triple artificial pollination enhanced pollen tube elongation and promoted ovary expansion (Figs 4–6).

In the greenhouse located in northern China, Indian jujube N blooms after late June, while wild Chinese jujube S and Chinese jujube D bloom from mid-April to mid-June. This creates natural temporal isolation between them [20]. Hu [17] attempted to overcome this barrier by applying cold-stored Chinese jujube pollen to Indian jujube; however, pollen viability declined sharply after 2 months of storage, and the interspecific crosses failed. This problem has remained unresolved for over two decades [17]. In this study, we developed an integrated strategy by combining temperature control with heavy pruning, which advanced the flowering time of Indian jujube N to April, achieving synchronization with Wild Chinese jujube S and Chinese jujube D under greenhouse conditions. This breakthrough eliminates the phenological mismatch between Chinese jujube and Indian jujube, providing a practical platform for interspecific hybridization in the genus *Ziziphu*s. Temperature is the primary mechanism that regulates the flowering process [24]. Low temperature can promote flowering in plants, a phenomenon known as cold stress-induced flowering [37]. Chen *et al.* [38] demonstrated that temperature stress could alter anthesis in perennial species. Dong *et al.* [39] further showed in lotus that specific low-temperature treatments break dormancy and rapidly activate floral primordia. In this study, a low-temperature treatment (1°C–18°C), which was applied over 2 months during winter in the greenhouse, advanced the initial flowering time of Indian jujube N from June to April, effectively synchronizing it with the flowering time of Chinese jujube.

In 1962, Kanta *et al.* achieved the first successful *in vitro* fertilization in *Papaver somniferum* [40]. Subsequent studies in *Dianthus* and *Nicotiana* produced normal hybrid embryos that developed into flowering plants [41]. Qi and Liu [42] pioneered embryo-rescue culture, increasing the seedling rate of Chinese jujube D × wild Chinese jujube S crosses from <5% to 38%. However, a complete protocol integrating AS and AC with ovary and embryo culture has not been reported in genus *Ziziphus* Mill.. The small jujube flowers possess a fused disc-and-stamen ring; field emasculation results in nearly 100% floral abscission. In this study, by synchronizing a temperature- and pruning-based regime, we advanced the anthesis of Indian jujube N by over 2 months, thereby aligning its flowering time with that of wild Chinese jujube S and Chinese jujube D. Sterile *in vitro* flowers were then artificially pollinated three times, yielding a fruit set exceeding 10%, with the highest reaching over 68%. This approach enables rapid introgression of disease-resistance and stress-tolerance alleles from Indian jujube into elite Chinese jujube cultivars without transgenesis, circumventing the lack of a stable transformation system [15] and shifting jujube breeding from empirical field crosses to precision *in vitro* engineering.

## Materials and methods

### Plant materials

All the three genotypes belonging to two species of genus *Ziziphus* Mill., a temperate species Chinese jujube (*Z. jujuba* Mill., 2*n* = 2*x* = 24) and a tropical species Indian jujube (*Ziziphus mauritiana* Lam., 2*n* = 4*x* = 48), were planted in the greenhouse. The two genotypes of Chinese jujube include cultivated wild ‘Suanzao’ (briefly S) and ‘Dongzao’ (briefly D). The Indian jujube used is a cultivated genotype ‘Niunaidaqingzao’ (briefly N). Indian jujube N is genetically more distant from Chinese jujube compared to the distance between Wild Chinese jujube S and Chinese jujube D [14].

### Plant materials introduction and acclimatization

In early March 2024, 20 three-year-old healthy trees of Wild Chinese jujube S and Chinese jujube D were selected from the nursery at the Shandong Binzhou National Agricultural Science and Technology Park, located 1.2 km east of Chenjia Village, Botou Town, Zhanhua District, Binzhou, Shandong, China. On 1 April 2024, the trees were carefully excavated with intact root balls, dipped in a hydrogel slurry, and transported to the experimental greenhouse. Forty-eight vigorous 3-year-old Indian jujube N trees (ground diameter, 3.5 ± 0.4 cm) were selected from Dong Sheng West Street, Meitian, Zhongluotan Town, Baiyun District, Guangzhou, Guangdong, China. These plants had been grown since the seedling stage in nonwoven fabric grow bags (40 cm in diameter × 35 cm in height). Prior to transport, the trunks were pruned to a height of 1.2 m, and the entire plants were wrapped in plastic film. The plants arrived at the Shandong Binzhou National Agricultural Science and Technology Park on 30 March 2024.

### Flowering-time regime

Cultivation environment: A 12 m × 70 m, east–west–oriented, winter-heated greenhouse (Greenhouse 1) was constructed in early March 2024 at the Shandong Binzhou National Agricultural Science and Technology Park. The soil inside is flat, well-drained, alkaline (pH 7.5–8.3), and moderately saline (3.3‰–4.2‰). On 1 April 2024, 20 wild Chinese jujube S, 20 Chinese jujube D, and 80 Indian jujube N trees were transplanted at equal intervals within the greenhouse 1; survival rate reached 95% that year. An additional 12 Indian jujube N trees were placed in Greenhouse 2, of which 11 survived. Except for temperature control and pruning treatments, soil conditions and routine management practices were identical between the two greenhouses. Primary branches arising from the trunk were manually bent, when morphologically appropriate, to an angle of 100°–150° relative to the trunk.

Temperature control: By adjusting the front and roof vents and deploying or retracting the overhead shade screen, we precisely controlled the temperature in Greenhouse 1. From 1 December 2024 to 25 February 2025, the minimum–maximum temperature was maintained at 1°C–18°C; from 26 February to 13 March 2025, at 10°C–22°C; and from 14 March to 1 June 2025, at 16°C–24°C. From December 2024 to June 2025, Greenhouse 2 maintained a temperature range of 8°C–32°C.

From the surviving Indian jujube N trees, uniform and vigorous trees were selected for canopy manipulation based on the following criteria: a single straight trunk terminating at ~1.2 m above ground level, more than eight primary branches arising from the trunk, and each bearing at least one secondary branch longer than 40 cm.

Treatment A: Five to six evenly distributed primary branches were retained and cut back to 1.2 m; all other primary branches and the secondary branches on the retained primary branches were removed.

Treatment B: This is identical to Treatment A, except that one secondary branch was retained on each of primary branches.

Treatment C (CK1): No pruning was applied.

Treatment D (CK2): No pruning was applied, and temperature control was not used.

Treatments A, B, and C were implemented in Greenhouse 1, while Treatment D was conducted in Greenhouse 2.

Treatments A and B were carried out on 26 February 2025 (Feb-A and Feb-B), 14 March 2025 (Mar-A and Mar-B), and 1 April 2025 (Apr-A and Apr-B). The tested trees were randomly assigned to seven pruning treatments (three trees per treatment). Each treatment was replicated three times in a completely randomized block design. Daily flower counts were averaged per treatment, rounded to the nearest integer, and visualized as a flowering heatmap using R v4.4.2.

### Pollen collection and storage

A randomized block design was used. Three trees per genotype (wild Chinese jujube S, Chinese jujube D, Indian jujube N) were randomly selected. One hundred mature, disease-free flowers per tree were gently detached using forceps, immediately placed in an ice-packed insulated container, and transported to the laboratory. Samples were stored at 4°C and used for pollen viability and germination assays within 24 h. Mature pollen was collected from *in vitro* cultured flowers under sterile conditions, transferred into fully sterilized zip-lock bags, stored at 4°C, and used for pollination within 24 h.

### Pollen viability assessment

Dissolve 2 g of KI in 5–10 ml of distilled water, add 1 g of I₂, and bring the volume to 200 ml after complete dissolution. Store the I₂-KI solution in an amber bottle. Using forceps, detach two to three anthers from freshly opened flowers of each genotype onto a microscope slide, add one drop of I₂-KI solution, mix thoroughly with a dissecting needle, and cover with a coverslip. Observe under a microscope and record staining: deep blue staining indicates viable pollen; pale or colorless grains indicate nonviable pollen. Repeat each species in triplicate; examine five random fields per replicate. Viability (%) = (deep blue stained grains/total grains) × 100.

### Pollen germination assay

The germination medium consisted of Murashige and Skoog (MS) salts, 6 g/l agar, 75 mg/l boric acid, and 30 g/l sucrose. After autoclaving and pouring into Petri dishes, the medium was allowed to solidify. Freshly collected pollen was evenly spread onto the surface and incubated at 25°C for 6 h. Pollen grains producing tubes longer than their diameter were scored as germinated. Each genotype was replicated three times; five random microscopic fields per replicate were examined, with at least 60 grains counted per field. Germination (%) = (germinated grains/total grains) × 100.

### *In vitro* culture of parental flowers

The medium formulation consisted of 1/2 MS, 50 g/l sucrose, 5.5 g/l agar, 0.1 g/l boric acid, 1 g/l activated charcoal, 0.5 mg/l Gibberellic Acid (GA), and 0.1 g/l Vitamin C (Vc).

#### Self-pollination

Each genotype received five treatments: SSP, ASP, ASC, ASS, and TAS (Table 1). The experiment followed a completely randomized block design, with three replicates per treatment. Each treatment consisted of 18 healthy, pest-free flowers at the yellow-bud stage. Flowers were cultured with one flower plus one retained leaf for wild Chinese jujube S and Chinese jujube D, and one flower plus half a leaf for Indian jujube N; flowers were allowed to open naturally without emasculation.

#### Cross-pollination

This experiment also followed a completely randomized block design. All pairwise interspecific combinations were generated (six crosses). Each cross included four treatments, ACP, ACC, ACS, and TAC (Table 1), arranged in three replicates. For each treatment, 18 healthy, pest-free flowers at the yellow-bud stage were collected. Maternal flowers were emasculated at the petal-crack stage immediately before pollination.

### Aniline blue staining, microscopic observation, and ovary expansion determination

Pollen tube targeting in ovules was assessed by using aniline blue staining, following a previously described methodology [15]. Briefly, ~48 h after pollination, pistils were harvested, trimmed, and immersed in a 1:3 (v/v) acetic acid–ethanol solution for at least 30 min. They were then rehydrated through a stepwise ethanol series (70%, 50%, and 30%) followed by distilled water, softened overnight in 8 mol/l NaOH, rinsed three times with distilled water, and stained for 2 h in the dark with 0.1% decolorized aniline blue prepared in 108 mM K_3_PO_4_. Observations and imaging were performed using a fluorescence microscope (Olympus BX43). Ovary expansion was evaluated 5 days after pollination; an ovary was classified as expanded only if its equatorial diameter had increased by ≥20% and measured ≥3 mm, its protrusion height was ≥2 mm, and it remained green and turgid. Any ovary failing to meet these criteria or showing shrinkage or yellowing was recorded as nonexpanded. Expansion rate (%) = (number of expanded ovaries/total flowers pollinated) × 100.

## Supplementary Material

Web_Material_uhag066

## Data Availability

All data required to evaluate the conclusions in this study are provided in the Supplementary material Table S1-S7.
