# Supplementary material for: Dual regime of flowering time and pollination enhances pollen tube development in Ziziphus
Source: Hortic Res. 2026 Mar 7;13(5):uhag066. doi: 10.1093/hr/uhag066 (PMC13175040; doi:10.1093/hr/uhag066)
Supplement: Web_Material_uhag066 [file web_material_uhag066.zip › Supplementary Data Table.docx]

Supplementary Data Table S1. Number of bud dehiscence

| Truncation date of Niunaidaqingzao | | | | | | | Truncation date of Suanzao | Truncation date of Dongzao |
| --- | --- | --- | --- | --- | --- | --- | --- | --- |
| 2025/2/26 | | 2025/3/14 | | 2025/4/1 | | |  |  |
| A | B | A | B | A | B | C |  |  |
| 0 | 0 | 0 | 0 | 0 | 0 | 0 | 0 | 5 |
| 0 | 0 | 0 | 0 | 0 | 0 | 0 | 1 | 2 |
| 0 | 2 | 0 | 0 | 0 | 0 | 0 | 0 | 3 |
| 0 | 1 | 0 | 0 | 0 | 0 | 0 | 0 | 6 |
| 0 | 5 | 0 | 0 | 0 | 0 | 0 | 2 | 0 |
| 0 | 7 | 0 | 0 | 0 | 0 | 0 | 4 | 2 |
| 0 | 8 | 0 | 0 | 0 | 0 | 0 | 8 | 4 |
| 0 | 13 | 0 | 0 | 0 | 0 | 0 | 11 | 11 |
| 0 | 17 | 0 | 0 | 0 | 0 | 0 | 8 | 9 |
| 0 | 14 | 0 | 3 | 0 | 0 | 0 | 3 | 6 |
| 0 | 13 | 0 | 1 | 0 | 0 | 0 | 16 | 13 |
| 0 | 11 | 0 | 6 | 0 | 0 | 0 | 12 | 18 |
| 0 | 10 | 0 | 2 | 0 | 0 | 0 | 17 | 16 |
| 0 | 15 | 0 | 12 | 0 | 0 | 0 | 16 | 22 |
| 0 | 16 | 0 | 16 | 0 | 1 | 0 | 14 | 14 |
| 0 | 25 | 0 | 20 | 0 | 4 | 0 | 22 | 18 |
| 0 | 32 | 0 | 18 | 0 | 11 | 0 | 21 | 25 |
| 0 | 36 | 0 | 27 | 0 | 16 | 2 | 26 | 27 |
| 0 | 20 | 0 | 30 | 0 | 12 | 6 | 31 | 23 |
| 0 | 25 | 0 | 36 | 0 | 22 | 0 | 28 | 31 |
| 0 | 28 | 0 | 42 | 0 | 18 | 7 | 35 | 35 |
| 0 | 23 | 0 | 32 | 0 | 26 | 4 | 30 | 38 |
| 1 | 25 | 0 | 28 | 0 | 31 | 11 | 28 | 34 |
| 0 | 32 | 0 | 34 | 0 | 42 | 16 | 24 | 37 |
| 3 | 41 | 0 | 27 | 0 | 38 | 18 | 31 | 51 |
| 2 | 36 | 3 | 22 | 0 | 26 | 28 | 34 | 55 |
| 6 | 43 | 0 | 27 | 0 | 23 | 31 | 42 | 56 |
| 0 | 37 | 0 | 26 | 0 | 14 | 26 | 38 | 42 |
| 16 | 22 | 6 | 42 | 0 | 26 | 24 | 49 | 46 |
| 15 | 17 | 2 | 49 | 0 | 31 | 36 | 51 | 37 |
| 22 | 13 | 5 | 41 | 0 | 46 | 31 | 47 | 32 |
| 20 | 10 | 17 | 52 | 0 | 55 | 38 | 42 | 36 |
| 19 | 13 | 12 | 46 | 2 | 51 | 42 | 40 | 31 |
| 34 | 10 | 16 | 31 | 6 | 60 | 49 | 27 | 22 |
| 37 | 13 | 23 | 24 | 12 | 45 | 51 | 35 | 16 |
| 46 | 8 | 19 | 16 | 27 | 34 | 59 | 24 | 7 |
| 55 | 0 | 25 | 3 | 39 | 27 | 41 | 30 | 11 |
| 37 | 2 | 31 | 14 | 34 | 23 | 46 | 16 | 15 |
| 40 | 0 | 36 | 6 | 48 | 18 | 28 | 22 | 5 |
| 26 | 0 | 61 | 3 | 56 | 6 | 16 | 11 | 1 |
| 31 | 4 | 46 | 2 | 50 | 2 | 9 | 5 | 0 |
| 21 | 6 | 51 | 0 | 38 | 9 | 7 | 6 | 7 |
| 16 | 0 | 46 | 0 | 37 | 1 | 12 | 0 | 3 |
| 22 | 2 | 30 | 0 | 27 | 3 | 4 | 6 | 0 |
| 13 | 0 | 16 | 0 | 31 | 0 | 0 | 3 | 0 |
| 8 | 0 | 10 | 0 | 23 | 5 | 0 | 0 | 5 |
| 2 | 0 | 0 | 0 | 36 | 2 | 0 | 0 | 2 |
| 0 | 0 | 6 | 0 | 19 | 0 | 3 | 4 | 0 |
| 3 | 0 | 3 | 0 | 8 | 0 | 0 | 8 | 0 |
| 5 | 0 | 0 | 0 | 2 | 0 | 2 | 3 | 0 |
| 0 | 0 | 2 | 0 | 5 | 0 | 0 | 0 | 0 |
| 0 | 0 | 0 | 0 | 0 | 0 | 0 | 0 | 0 |
| 0 | 0 | 0 | 0 | 3 | 0 | 0 | 0 | 0 |
| 0 | 0 | 0 | 0 | 0 | 0 | 0 | 0 | 0 |
| 0 | 0 | 0 | 0 | 1 | 0 | 0 | 0 | 0 |

Supplementary Data Table S2. Number of high-viability pollen

| Taxon | Number of high-viability pollen | | |
| --- | --- | --- | --- |
| Suanzao | 67 | 57 | 61 |
| Dongzao | 37 | 43 | 45 |
| Niunaidaqingzao | 29 | 35 | 37 |

Supplementary Data Table S3. Number of germinated pollen grains

|  | | | |
| --- | --- | --- | --- |
| Taxon | Number of germinated pollen grains | | |
| Suanzao | 18 | 24 | 20 |
| Dongzao | 9 | 14 | 11 |
| Niunaidaqingzao | 13 | 18 | 17 |

Supplementary Data Table S4. Self-pollinated pollen tube elongation

|  | | | | | | | | | | | | | | | |
| --- | --- | --- | --- | --- | --- | --- | --- | --- | --- | --- | --- | --- | --- | --- | --- |
| Taxon | Number of samples for self-pollinated pollen tube elongation | | | | | | | | | | | | | | |
|  | SSP | | | ASP | | | ASC | | | ASS | | | AST | | |
| Suanzao | 7 | 9 | 6 | 0 | 0 | 0 | 10 | 12 | 12 | 9 | 7 | 10 | 14 | 17 | 16 |
| Dongzao | 3 | 4 | 3 | 0 | 0 | 0 | 5 | 7 | 9 | 5 | 4 | 3 | 11 | 11 | 10 |
| Niunaidaqingzao | 8 | 8 | 7 | 0 | 0 | 0 | 9 | 10 | 12 | 6 | 8 | 6 | 13 | 15 | 15 |

Supplementary Data Table S5. Self-pollination ovary expansion

|  | | | | | | | | | | | | | | | |
| --- | --- | --- | --- | --- | --- | --- | --- | --- | --- | --- | --- | --- | --- | --- | --- |
| Taxon | Number of samples for self-pollination ovary expansion | | | | | | | | | | | | | | |
|  |  | | | | | | | | | | | | | | |
|  | SSP | | | ASP | | | ASC | | | ASS | | | AST | | |
| Suanzao | 5 | 4 | 4 | 0 | 0 | 0 | 3 | 2 | 4 | 5 | 8 | 6 | 12 | 14 | 11 |
| Dongzao | 0 | 0 | 0 | 0 | 0 | 0 | 0 | 0 | 0 | 0 | 0 | 0 | 0 | 0 | 0 |
| Niunaidaqingzao | 2 | 3 | 2 | 0 | 0 | 0 | 3 | 2 | 3 | 2 | 1 | 3 | 6 | 4 | 5 |

Supplementary Data Table S6. Cross-pollinated pollen tube elongation

|  | | | | | | | | | | | | | |
| --- | --- | --- | --- | --- | --- | --- | --- | --- | --- | --- | --- | --- | --- |
| Hybrid combination | Number of samples for self-pollinated pollen tube elongation | | | | | | | | | | | |  |
|  | ACP | | | ACC | | | ACS | | | ACT | | |  |
| ♀ S × ♂ D | 0 | 0 | 0 | 5 | 6 | 6 | 6 | 3 | 4 | 8 | 10 | 9 |  |
| ♀ S × ♂ N | 0 | 0 | 0 | 8 | 5 | 6 | 6 | 5 | 4 | 10 | 10 | 9 |  |
| ♀ D × ♂ S | 0 | 0 | 0 | 11 | 9 | 9 | 11 | 8 | 8 | 14 | 13 | 12 |  |
| ♀ D × ♂ N | 0 | 0 | 0 | 6 | 9 | 7 | 5 | 7 | 6 | 9 | 7 | 13 |  |
| ♀ N × ♂ S | 0 | 0 | 0 | 11 | 7 | 12 | 8 | 11 | 7 | 13 | 9 | 13 |  |
| ♀ N × ♂ D | 0 | 0 | 0 | 3 | 6 | 7 | 7 | 7 | 5 | 7 | 5 | 10 |  |

Supplementary Data Table S7. Cross-pollination ovary expansion

| Hybrid combination | Number of samples for self-pollination ovary expansion | | | | | | | | | | | |
| --- | --- | --- | --- | --- | --- | --- | --- | --- | --- | --- | --- | --- |
|  | ACP | | | ACC | | | ACS | | | ACT | | |
| ♀ S × ♂ D | 0 | 0 | 0 | 0 | 0 | 0 | 0 | 0 | 0 | 0 | 0 | 0 |
| ♀ S × ♂ N | 0 | 0 | 0 | 5 | 3 | 4 | 4 | 4 | 3 | 8 | 7 | 6 |
| ♀ D × ♂ S | 0 | 0 | 0 | 3 | 2 | 3 | 3 | 4 | 3 | 5 | 5 | 4 |
| ♀ D × ♂ N | 0 | 0 | 0 | 4 | 6 | 4 | 4 | 6 | 5 | 7 | 5 | 8 |
| ♀ N × ♂ S | 0 | 0 | 0 | 6 | 4 | 7 | 5 | 6 | 5 | 8 | 7 | 7 |
| ♀ N × ♂ D | 0 | 0 | 0 | 0 | 0 | 0 | 0 | 0 | 0 | 0 | 0 | 0 |
